# Socioeconomic and academic consequences of COVID-19 pandemic on medical students from the University of Rwanda

**DOI:** 10.1371/journal.pone.0318066

**Published:** 2025-02-13

**Authors:** Olga Nadege Uwera Ndamukunda, Marie Therese Mutuyimana, Fabiola Umubano, Eugene Tuyishime

**Affiliations:** 1 College of Medicine and Health Sciences, School of Medicine and Pharmacy, University of Rwanda, Kigali, Rwanda; 2 Department of Anesthesia, Critical Care, and Emergency Medicine, University of Rwanda, Kigali, Rwanda; World Health Organization, SOUTH SUDAN

## Abstract

**Introduction:**

Little is known about the consequences of the COVID-19 pandemic on the life of university students in Sub Saharan Africa (SSA). The objective of this study was to evaluate the socioeconomic and academic consequences of the COVID-19 pandemic on medical students studying at the University of Rwanda.

**Methods:**

This was a cross-sectional study. An online survey using google form was sent to medical students in clinical training (year 3 till year 5) using convenience sampling followed by snowball sampling method. We collected data on participants’ demographics, general knowledge on the COVID-19 pandemic and perception on mitigation measures, and socio-economic and academic consequences of the COVID-19 pandemic. Descriptive statistics were used in excel 2015 software to calculate participants’ responses and categorical data were presented using frequencies and percentages.

**Results:**

A total 187 participants completed the survey. Most participants described disruption in routine activities (72.7%), reduced travelling (69%), church closing (64.2%), and loss of freedom (57.2%) as examples of negative social consequences. While financial uncertainty (64.7%), decrease in income (49.7%), and increase in poverty rate (42.2%) were the main economic consequences. Issues with academic progress (95.7%), limited social life (56.1%), and repeating the year (42.8%) were examples of negative academic consequences.

**Conclusion:**

The results of this study suggest that the COVID-19 had a negative social, economic, and academic consequences on medical students at the University of Rwanda. These finding may guide the design of interventions to mitigate the consequences of COVID-19 and to protect medical students against future pandemics and crises.

## Background

The Covid-19 pandemic has disrupted multiple sectors of the economy globally, but low-income countries were affected the most due to limited resources. Multiple negative socio-economic consequences have been described such as limited movement, job loss, financial instability, adverse effects on human communication and relationships, and suffering and feeling helpless, especially for vulnerable people like the poor, elderly, persons with disabilities, and homeless people [1–5]. Social life was severely affected with churches being closed; sports prohibited, and stadiums and gyms closed. As all leisure and relaxing places were closed to try to contain the pandemic, the fear of an uncertain future and the constant stress of illness caused intense psychological pressure that led to an increase in rates of depression, suicide, domestic violence (physical, emotional, or sexual), and other mental illnesses [2,6].

In addition, the COVID-19 caused specific socioeconomic and academic consequences to the education sector, especially in low resources settings. University students from different countries faced socio-economic challenges such as income loss, financial uncertainty, food insecurity, and mental health difficulties [1,5]. On top of that, university students faced academic consequences like missing classes, difficulty in academic progression, and repeating the academic year. This may have an even greater negative consequences on medical students who faced the dilemma to continue clinical rotations with the risk of exposure or taking time off clinical rotation with the risk of poor training and difficulty in academic progression [6–10]. This happened in the context of multiple consequences of the COVID-19 pandemic on key sectors of the Rwandan economy such as manufacturing, tourism, services, and agriculture; manufacturing faced supply chain disruptions, tourism suffered a big loss due to travel restrictions, most services were closed, and the agriculture output was reduced [11,12]. This led to the contraction of the economy by 3.4% in 2020 (first negative growth in the last 2 decades), however, Rwanda’s proactive measures facilitated a good recovery [11,12].

To our knowledge, there is no study which has been done in Rwanda to explore the socio-economic and academic consequences of the COVID-19 on medical students in Rwanda. To close this gap in knowledge, this study had the following objectives: 1) to evaluate the socio-economic consequences of the COVID-19 pandemic on medical students in clinical years at the University of Rwanda; 2) to evaluate the academic consequences of COVID-19 pandemic by medical students in clinical years at the University of Rwanda.

## Methods

### Study description

This was a cross-sectional study. Reporting followed the Strengthening the Reporting of Observational Studies in Epidemiology (STROBE) guidelines for reporting observational studies [13].

### Study settings and population

This study was conducted at the University of Rwanda (UR), in the College of Medicine and Health Sciences (CMHS), in the School of Medicine and Pharmacy, which had approximately 700 medical students. Medical students in clinical training (year 3 up to year 5) were eligible to participate in this study. We excluded participants who did not provide their informed consent.

### Data collection

Data were collected using an online survey (google form); the link was shared via WhatsApp and emails participants by convenience sampling followed by snowball recruitment method. Participants were requested to share the link to others so that it reaches as many participants as possible. The link was open on 20^th^ April 2022 and closed on 01^st^ July 2022.

The questionnaire had 4 groups of questions including demographic profile, general knowledge on COVID-19 pandemic, its socioeconomic consequences, and its academic consequences. The questionnaire was adapted from similar studies done in Pakistan and Liberia by respectively Ali et al. 2020 and Davis et al. 2021[1,6].

### Statistical analysis

We used descriptive statistics in excel 2015 software to calculate participants’ responses and categorical data were presented using frequencies and percentages. No continuous variables were reported.

### Ethical approval

Ethical approval was obtained from the College of Medicine and Health Sciences Institutional Review Board, IRB number: CMHS/IRB/242/2022. All participants provided written informed consent prior to participating in the study.

## Results

In total, 187 medical students participated in the survey. Most participants were male (52.9%), aged between 24-29 years (75.4%), stayed with parents (44.9%), come from Kigali (50.2%), and were in Ubudehe category 3 (83.9%) (Table 1).

**Table 1:** Sociodemographic characteristics.

Variables	Descriptions	Number	%
Age range (in years)	24–29	141	75.4
	29–35	26	13.9
	18–23	17	9.1
	>35	3	1.6
Sex	Male	99	47.1
	Female	88	52.9
Year of study	Doc2	96	51.3
	Doc3	66	35.3
	Doc1	11	5.9
	Preclinic 2	9	4.8
	Preclinic 1	5	2.7
Marital status	Single	159	85.5
	Married	26	14
	Divorced	1	0.5
Place of stay	With parents	84	44.9
	University’s hostel	60	32.1
	Tenant near the campus	41	21.9
	Live with relatives	1	0.5
Province of origin	Kigali city	87	50.2
	Western province	29	16.7
	Northern province	24	13.8
	Southern province	21	12.1
	Eastern province	12	6.9
Religion	Catholic	94	51.6
	Protestant	72	39.6
	Adventist	7	3.7
	Muslim	1	0.5
	Reformer	1	0.5
	None	7	3.8
Economic status of the family	Category 3	151	83.9
	Category 2	22	12.2
	Category 4	5	2.8
	Category 1	2	1.1

Table 2 describes participants’ general knowledge on COVID-19 pandemic and mitigation measures. Most participants were aware of COVID-19 pandemic (97.3%) and used appropriate mitigation measures like vaccination (89.3%), using face masks every day (82.9%), and washing their hands frequently (81.8%).

**Table 2:** General knowledge on COVID 19 pandemic of medical students from the University of Rwanda.

Variables	Descriptions	Number	%
Covid-19 awareness in Rwanda	Aware	182	97.3
	Not aware	5	2.7
Type of disease is COVID-19	Communicable	183	97.9
	Non communicable	2	1.1
	Simple disease	2	1.1
Believe in COVID-19 existence	Believe in Covid-19	178	95.2
	Don’t believe in Covid-19	9	4.8
COVID-19 in respective neighborhood	Believe it reached there	113	63.8
	It didn’t reach there	64	36.2
Reason to believe that COVID-19 pandemic is unreal	Political issue	8	25.8
	Simple flu not a pandemic	7	22.6
	A rumor	5	16.1
	Others *	11	35.2
Used protective measures to prevent COVID-19 spread	Vaccination	167	89.3
	Wearing mask	155	82.9
	Frequent handwashing	153	81.8
	Social distancing	115	61.5
	Avoiding unnecessary movements	111	59.4
	Stay at home	99	52.9
	Others**	4	2
Tested positive for COVID-19	Did not have COVID-19	149	79.7
	Tested positive	38	20.3

*Religious belief, low level of education, anti-vaccine views, misinformation.

**Regular testing, physical exercise, abiding to mitigation measures.

**Fig 1:**
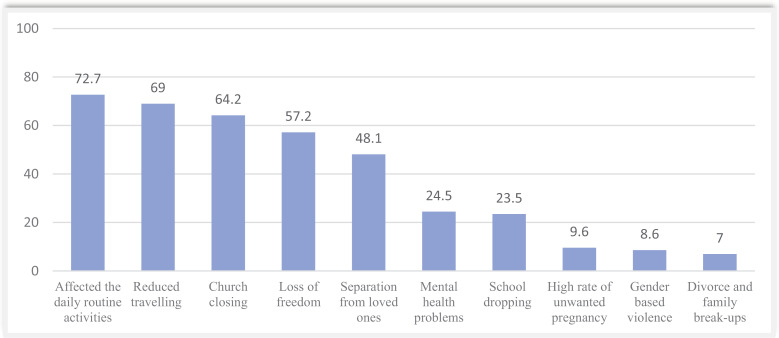
Social consequences of COVID-19 pandemic on medical students from the University of Rwanda.

Fig 1 shows the social consequences of COVID-19 pandemic among medical students at the University of Rwanda. Most participants faced disruption of routine activities such church closure (64.2%), restricted travelling (69%). Other negative social consequences reported include separation from loved ones (48.1%), loss of freedom (57.2%), and dropping out from school (23.5%).

Table 3 shows the economic consequence of the COVID-19 pandemic. 64.7% of participants said that financial uncertainty was the major immediate consequence of the COVID-19 on personal level, followed by a decrease in income at 49.7%, and fear of school dropout at 39%. At family level, 42.2% of participants showed that poverty is the main consequence while 38% faced business closure.

**Table 3:** Economic consequences of COVID-19 pandemic on medical students from the University of Rwanda.

Variables	Description	Number	%
Immediate consequences of COVID-19 pandemic on personal level	Financial uncertainty	121	64.7
	Decrease in income	93	49.7
	Fear of school drop out	73	39
	Food crisis	64	34.2
	Fear of missing school fees	60	32.1
	House eviction	19	10.2
	Others *	12	6.1
Immediate consequences of COVID-19 on one’s family	Poverty	79	42.2
	Business closure	71	38
	Unemployment	63	33.7
	Food crisis	54	28.9
	Dropping out of school	28	15
	House eviction	12	6.4
	None	5	2.7
	Others**	22	11.1
Importance of total lockdown in prevention of the spread of COVID-19	Moderately helpful	91	48.7
	Very Helpful	77	41.2
	Not helpful	19	10.2
Source of day-to day income and food supply during the total lockdown	Savings	97	51.9
	Government monthly allowance	41	21.9
	Part time job	15	8
	Return from investment	11	5.9
	Government support	6	3.2
	Others***	17	8.8

*None, delay in school progress, posponed academic activities.

**Decrease in income, financial insecurities, economic crisis, consequences *on other relatives.*

***Help from parents and family, personal business income

**Fig 2:**
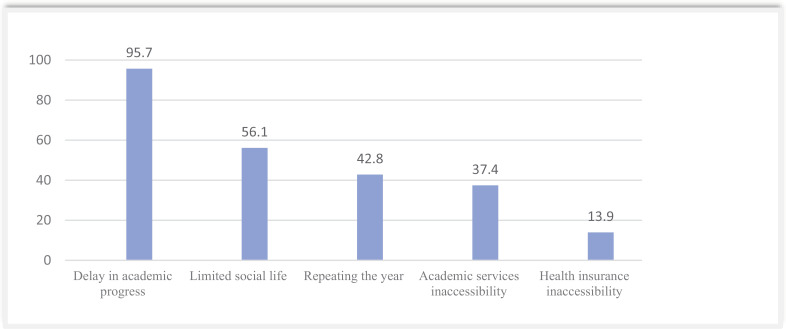
Academic consequences of the COVID-19 on medical students from the University of Rwanda.

Majority of participants showed their concern about academic progress (95.7%) and limited social life (56.1%) (Fig 2).

**Fig 3:**
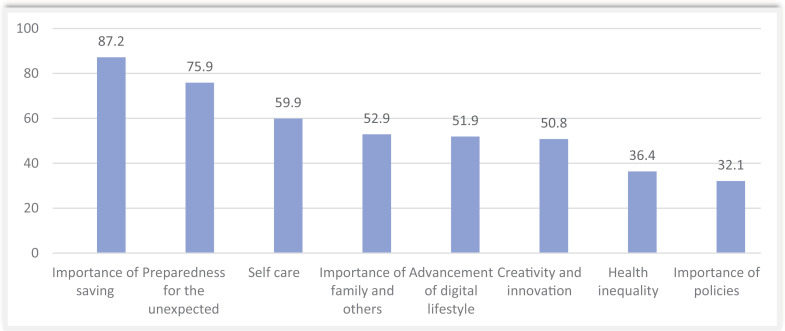
Lessons learnt from the COVID-19 consequences by medical students from the University of Rwanda.

Most participants understood the importance of saving (87.2%), learned to always be prepared for the unexpected (75.9%), learned the importance of self-care (59.9%), and value their family more than before (52.9%) (Fig 3).

## Discussion

The findings of this study showed that most participants faced different social consequences like disruption in routine activities (72.7%), reduced travelling (69%), church closing at (64.2%), and loss of freedom (57.2%). Those impacts were mainly attributed to the mitigation measures taken to contain the spread of the virus, specifically total lockdowns, and quarantines. Similar findings have been described in other countries.

For example, Amjad Ali et al., 2020 found that COVID-19 led to negative social consequences on people living in Gilgit-Baltistan mountains in Pakistan such as routine activities disturbance, limited social life, and reduced traveling [1]. Similarly, Tewodros Mulugeta et al., 2021 found similar findings in Addis Ababa/Ethiopia with most participants expressing how it was unbearable to stay at home leading to stress, boredom, and anxiety [2]. Furthermore, Busetta et al., 2021 showed that movements restriction has deeply impacted the social life of university students in Italy, with increased rate of anxiety and future uncertainty, however, that experience opened their eyes on a different aspect of university life specifically e-learning [14]. In Greece, K. Diamanti et al., 2021 showed that many students experienced social deprivation contacting only their parents and family members leading to a loss of live communication, isolation feeling, loss of freedom, and lack of personal space when at home. However, this study showed some positive impact like strengthening family relations [15].

Despite all the negative consequences experienced during the pandemic crisis, we cannot ignore the positive side it had on families. Alghamdi A.A., 2021 during the survey of Saudi university students; participants described that the COVID-19 pandemic helped them to build strong bond and connection with their families, to increase appreciation of life and death, and to set priorities [9]. Being at home maybe suffocating and depriving but it was proven that it is also an important element to rebuild family’s bond and communication. Parents should focus more on open communication with their children and spend enough time with them for their social and mental wellbeing.

Most participants reported financial uncertainty (64.7%), decrease in income (49.7%), and increase in poverty rate (42.2%) as the main economic consequences. Multiple studies have reported similar findings.

For example, Elvis et al., 2021 found that income loss, financial uncertainty and food insecurity were major burden faced by medical and pharmacy students in Liberia [6]. Cvetkovic et al., 2020 found that unemployment and fear of job loss were the major consequences among university students in Serbia [3]. Also, some countries faced limited economic consequences. For example, Wendy et al., 2021, showed that rural households in Kenya faced income decline, however, the food security was intact because people were consuming their home-grown foods which is an advantage for countries focusing on agriculture as a major source of income [16].

Our findings of high rates of reporting negative socioeconomic consequences may be explained by the fact that most medical students from the University of Rwanda come from low-income families living far from the University. Medical students may have had difficulties in getting financial and social support from their families. In addition, the government stipend even if it is helpful but it is still small. There are also limited opportunities for part-time jobs. More social protection measures for university students should be put in place at student level, family level, and government level.

The academic life was also impacted by the pandemic, as the study showed that 95.7% faced delay in the academic progress, 56.1% complained of limited social life and 42.8% repeated the year.

Similar findings have been described in the literature. For example, Maria et al., 2020 showed that university students in Bacau, Romania faced limited social life and poor communication with their lecturers affecting their accessibility to learning materials and mental health degradation [8]. Halis Sakiz, 2022 study on individuals with disability in Turkey showed that the pandemic caused significant delay in academic progress, poor access to mental health services and lack of socialization.

In contrast, Gonzalez et al., 2020 showed the positive impact of the COVID-19 pandemic on university students in Spain; their workload was not affected, the online study increased the assessment activities, and the learning strategy became more regular than before [17].

In the context of Rwanda, our findings of reporting high rates of negative academic consequences may be explained by lack of enough experience in using e-learning which was also limited by lack of access to high speed interned and computers for some students. Initiatives to promote easy access to online courses for medical students at the University of Rwanda should be encouraged.

### Limitations

This study has different limitations to consider. First, this study was conducted in one medical school in Rwanda and used convenience sampling method; the results may not be generalizable to other medical schools in Rwanda with different characteristics or other countries with different context. In addition, our survey was distributed through WhatsApp and emails requiring access to internet, we may have missed some participants in rural areas without internet who may even have had higher negative socioeconomic and academic consequences. Moreover, there is a possibility of recall bias leading to under-reporting some consequences of the COVID-19 pandemic among some students. Finally, our analysis used only descriptive statistics which did not show factors associated with more consequences from the COVID-19 pandemic.

## Conclusion

The results of this study suggest that the COVID-19 had a negative social, economic, and academic consequences on medical students at the University of Rwanda. These finding may guide the design of interventions to mitigate the consequences of the COVID-19 pandemic and to protect medical students against future pandemics and crises. Further studies should evaluate the feasibilities of potential interventions to improve pandemic preparedness among medical students in Rwanda.

## Supporting information

S1 File Data collection tool. (XLSX)

## References

[1] Ali A, Ahmed M, Hassan N. Socioeconomic impact of COVID-19 pandemic: evidence from rural mountain community in Pakistan. J Public Aff. 2021 Nov;21(4):e2355. doi: 10.1002/pa.2355 32904946 PMC7460993

[2] Mulugeta T, Tadesse E, Shegute T, Desta TT. COVID-19: socio-economic impacts and challenges in the working group. Heliyon. 2021;7(6):e07307. doi: 10.1016/j.heliyon.2021.e07307 34151042 PMC8196481

[3] Cvetković VM, Nikolić N, Nenadić UR, Öcal A, Noji EK, Zečević M. Preparedness and preventive behaviors for a pandemic disaster caused by COVID-19 in Serbia. Int J Environ Res Public Health. 2020;17(11):1–23. doi: 10.3390/ijerph17114124 32527056 PMC7313005

[4] Osterrieder A, Cuman G, Pan-Ngum W, Cheah PK, Cheah PK, Peerawaranun P, et al. Economic and social impacts of COVID-19 and public health measures: results from an anonymous online survey in Thailand, Malaysia, the UK, Italy and Slovenia. BMJ Open. 2021;11(7):e046863. doi: 10.1136/bmjopen-2020-046863 34285007 PMC8295020

[5] Plan ER. The socio-economic impact of COVID-19 in Rwanda. 2020.

[6] Davis EJ, Amorim G, Dahn B, Moon TD. Perceived ability to comply with national COVID-19 mitigation strategies and their impact on household finances, food security, and mental well-being of medical and pharmacy students in Liberia. PLoS One. 2021;16(7):1–13. doi: 10.1371/journal.pone.0254446 34242378 PMC8270202

[7] Aristovnik A, Keržič D, Ravšelj D, Tomaževič N, Umek L. Impacts of the COVID-19 pandemic on life of higher education students: global survey dataset from the first wave. Data Brief. 2021;39:107659. doi: 10.1016/j.dib.2021.107659 34869802 PMC8634691

[8] Radu MC, Schnakovszky C, Herghelegiu E, Ciubotariu VA, Cristea I. The impact of the covid-19 pandemic on the quality of educational process: a student survey. Int J Environ Res Public Health. 2020;17(21):7770. doi: 10.3390/ijerph17217770 33114192 PMC7660608

[9] Alghamdi AA. Impact of the COVID-19 pandemic on the social and educational aspects of Saudi university students’ lives. PLoS One. 2021;16(4):e0250026. doi: 10.1371/journal.pone.0250026 33852627 PMC8046245

[10] Mukherjee S. Emerging infectious diseases: epidemiological perspective. Ind J Dermatol. 2017;62:459–67. doi: 10.4103/ijd.IJD_379_17 28979007 PMC5618832

[11] UNDP. 2020. [cited 2024 Dec 12]. Available from: https://www.undp.org/sites/g/files/zskgke326/files/migration/africa/UNDP-rba-COVID-assessment-Rwanda.pdf

[12] NISR. 2020. [cited 2024 Dec 12]. Available from: https://www.statistics.gov.rw/publication/2020-rwandas-gdp-drop-34-following-covid-19-pandemic-outbreak

[13] von Elm E, Altman DG, Egger M, Pocock SJ, Gøtzsche PC, Vandenbroucke JP; STROBE Initiative. The strengthening the reporting of observational studies in epidemiology (STROBE) statement: guidelines for reporting observational studies. Prev Med. 2007 Oct;45(4):247–51. doi: 10.1016/j.ypmed.2007.08.012 17950122

[14] Busetta G, Campolo MG, Fiorillo F, Pagani L, Panarello D, Augello V. Effects of COVID-19 lockdown on university students’ anxiety disorder in Italy. Genus. 2021;77(1):1–16. doi: 10.1186/S41118-021-00135-5/figures/1 34658399 PMC8502092

[15] Diamanti K, Nikolaou SM. Researching the social impact of the COVID-19 pandemic on students in Greece. Eur J Dev Stud. 2021;1(3):26–32. doi: 10.24018/ejdevelop.2021.1.3.35

[16] Janssens W, Pradhan M, de Groot R, Sidze E, Donfouet HPP, Abajobir A. The short-term economic effects of COVID-19 on low-income households in rural Kenya: an analysis using weekly financial household data. World Dev. 2021;138. doi: 10.1016/j.worlddev.2020.105280

[17] Gonzalez T, De la Rubia MA, Hincz KP, Subirats L, Fort S, Sacha GM. Influence of COVID-19 confinement on students’ performance in higher education. PLoS One. 2020;15(10):e0239490. doi: 10.1371/journal.pone.0239490 33035228 PMC7546684

